# Genetic Polymorphisms of Vitamin D Receptor and Immune-Metabolic Mechanisms in Recurrent Pregnancy Loss: Narrative Review

**DOI:** 10.3390/biology15110817

**Published:** 2026-05-22

**Authors:** Gulzhanat Aimagambetova, Rita Nemr, Kuralay Atageldiyeva, Wassim Y. Almawi

**Affiliations:** 1 Department of Surgery, School of Medicine, Nazarbayev University, Astana 010000, Kazakhstan; 2 Clinical Academic Department of Women’s Health, CF University Medical Center, Astana 010000, Kazakhstan; 3 Department of Endocrinology, Lebanese American University Medical Center-Rizk Hospital (LAUMCRH), Beirut P.O. Box 11-3288, Lebanon; ritanemr@gmail.com; 4 Department of Medicine, School of Medicine, Nazarbayev University, Astana 010000, Kazakhstan; kuralay.atageldiyeva@nu.edu.kz; 5 Faculté des Sciences, Université de Tunis El Manar, Tunis 2092, Tunisia; wassim.almawi@fst.utm.tn

**Keywords:** miscarriage, recurrent pregnancy loss, immune tolerance, polymorphism, vitamin D, vitamin D receptor, *VDR*

## Abstract

Recurrent pregnancy loss (RPL) is a multifactorial reproductive disorder with important genetic, endocrine, immune, and metabolic determinants. Growing evidence links vitamin D deficiency and vitamin D receptor (*VDR*) gene polymorphisms to pregnancy complications, including RPL. A narrative review was conducted via a literature search in major databases: PubMed, EMBASE, Scopus, and Web of Science from January 2010 to January 2026, which synthesized observational studies on maternal 25-hydroxyvitamin D [25(OH)D] status and/or *VDR* polymorphisms in RPL, with predefined eligibility criteria and qualitative risk-of-bias assessment. Most studies focused on FokI (rs2228570) and the 3′ regulatory block BsmI/ApaI/TaqI. FokI is the most extensively studied VDR variant in RPL and showed the most consistent directional association compared with other variants, particularly in Asian and Middle Eastern populations, though the effect varied by study design, ancestry, and covariate adjustment. Contrary to that, investigations of BsmI/ApaI/TaqI loci were not consistent due to ancestry-specific linkage disequilibrium (LD). Genotype and vitamin D interaction effects were scarcely studied, with stratified analyses indicating a more significant genotype effect under vitamin D deficiency. From clinical practice perspectives, *VDR* polymorphisms may explain why some patients with RPL have a poor response to standard vitamin D supplementation. Women with the FokI f allele polymorphism associated with lower VDR activity require higher vitamin D dosing or earlier immunomodulatory support to achieve adequate endometrial receptivity. VDR variation, particularly FokI, may contribute to RPL susceptibility within an endocrine–immune–metabolic framework. Clinical translation will require standardized RPL definitions, concurrent 25(OH)D assessment, robust control for confounders, and analytical models that account for gene–environment interactions.

## Simple Summary

Recurrent pregnancy loss (RPL) is a complex reproductive system disorder associated with multiple factors, including genetics, immunity, and metabolism. According to recent studies, different gene variations, including those associated with vitamin D receptors, may be involved in the regulation of immunity and the development of pregnancy complications. This paper focuses on the investigation of the effect of genetic polymorphisms in contributing to the immune/metabolic imbalance in patients with recurrent pregnancy loss. The results obtained through the study can be used in assessing the risks of the disease occurrence and devising novel treatment and prevention approaches for RPL.

## 1. Introduction

Recurrent pregnancy loss (RPL) is a common and clinically significant complication of early reproduction, affecting 1–5% of women of reproductive age, and remains a major clinical challenge [1]. However, prevalence estimates vary because professional societies define RPL differently, creating diagnostic and research heterogeneity, including in genetic studies [1,2]. The American Society for Reproductive Medicine (ASRM) and European Society of Human Reproduction and Embryology (ESHRE) define RPL as two or more failed clinical pregnancies, whereas the Royal College of Obstetricians and Gynecologists (RCOG) traditionally requires three losses and differs on including biochemical pregnancies or gestational-age criteria [3,4]. Additional variation in the prevalence results from the inclusion of biochemical pregnancies, ectopic pregnancies, gestational age limits, and distinction between primary versus secondary RPL, in turn affects the reported prevalence, study comparability, and interpretation of risk-factor associations. Clinically, the diagnosis of RPL relies on history, examination, and targeted investigations [2,3,4]. Early losses (<10 weeks) often reflect chromosomal, endocrine, or metabolic causes, while later losses more commonly involve uterine anomalies, coagulation disorders, or cervical insufficiency [1,5]. Risk increases with maternal age and each additional loss [2,6]. Other contributors include immune, endocrine, genetic, and lifestyle factors [2,7,8]. Nevertheless, over 50% of RPL cases remain unexplained [2,9], necessitating further investigations to identify possible causative factors such as oxidative stress, epigenetic changes, and metabolic dysfunction.

Vitamin D deficiency is increasingly recognized as a common and potentially modifiable factor in women with repeated pregnancy loss. Furthermore, insulin resistance is associated with female reproductive problems and is likely to impact negatively on endometrial receptivity and early placental development [10]. Obesity can exacerbate the risk through the induction of chronic inflammation and alterations in the intrauterine environment [11,12]. Dyslipidemia, defined as elevated low-density lipoprotein (LDL) and triglycerides with reduced high-density lipoprotein (HDL), has been linked to impaired decidualization and vascular dysfunction [12,13]. It was also reported that altered amino acid metabolism, an imbalance between folate and homocysteine, fatty-acid-driven oxidative stress, and impaired vitamin D synthesis/signaling [14,15] pathways are closely connected to vitamin D biology.

Vitamin D (25-hydroxyvitamin D [25(OH)D]) supports implantation, chorionic development, placentation, and immune tolerance [16]. Its deficiency is associated with systemic endocrine and metabolic disorders [14,17], including insulin resistance, obesity, inflammation, and immune dysregulation [16,18], suggesting a possible contribution to impaired implantation, placentation, and maternal–fetal immune tolerance [19,20]. The most frequently investigated polymorphisms in the vitamin D receptor (*VDR*) include FokI (rs2228570), BsmI (rs1544410), ApaI (rs7975232), and TaqI (rs731236). Among these, FokI has shown the most reproducible association with RPL in several Asian and Middle Eastern cohorts. In contrast, the association of the other common vitamin D receptor (VDR) polymorphisms BsmI, ApaI, and TaqI with altered RPL risk shows inconsistent results [21], likely due to differences in ethnicity, sample size, RPL definitions, and unmeasured factors such as vitamin D status [22]. An additional challenge in interpreting this literature is that many candidate-gene studies of VDR variants in RPL were conducted in small cohorts, frequently involving fewer than 200 participants, which limits statistical power and reproducibility. This review synthesizes evidence on the *VDR* gene variants within the broader endocrine–immune–metabolic context underlying the implantation process and early placentation events, highlighting their potential role in RPL susceptibility.

Contrasting earlier reviews that addressed vitamin D deficiency or isolated *VDR* polymorphisms separately [23,24,25,26], this review combines puzzles of epidemiological data with evidence from genetic association and metabolic pathway studies to create a clear picture, allowing for the evaluation of vitamin D deficiency and VDR variants’ contribution to RPL. The unique aspect of this review is the integration of functional polymorphisms in the vitamin D receptor, regulatory polymorphisms, and endocrine–immune interactions within one mechanistic model that pertains to implantation and early placentation. Inconsistencies in previous research have been explained as a result of differences in phenotypes, population-specific linkage disequilibria, and environmental interactions that have not been accounted for in the past, stressing that the determination of VDR genotypes without considering 25(OH)D levels is not enough in predicting RPL.

## 2. Literature Search and Study Selection

Despite increasing attention being paid to the effects of vitamin D on fertilization and the role of its deficiency in infertility, data continue to remain dispersed between clinical trials, molecular studies, and genetics. Vitamin D insufficiency, immune system changes, VDR malfunction, or the *VDR* gene polymorphism are studied individually in research papers, and their synergistic impact on RPL remains unknown. The lack of an integrated approach that could provide a mechanism for explaining how hormonal insufficiency and genetic factors contribute to implantation and mother-to-child immunologic acceptance is evident.

This structured narrative literature review was performed based on a comprehensive search of relevant publications in research databases such as PubMed, EMBASE, Scopus, and Web of Science. The following inclusion criteria were applied: (1) studies published between January 2010 and January 2026 (2) in English (3) describing human research were identified as eligible. Articles published before 2010 in other languages and describing non-human research were excluded. The search strategy used the Boolean threads for combined terms related to RPL and vitamin D/*VDR* genetics: (“recurrent pregnancy loss” OR “recurrent miscarriage” OR “recurrent spontaneous abortion” OR “habitual abortion”) AND (“vitamin D” OR “25-hydroxyvitamin D” OR “25(OH)D”) AND (“vitamin D receptor” OR “VDR” OR “VDR polymorphism” OR “VDR gene” OR “FokI” OR “BsmI” OR “ApaI” OR “TaqI” OR rs2228570 OR rs1544410 OR rs7975232 OR rs731236). As definitional heterogeneity likely influences comparability, the operational definition of RPL used in each study (≥2 losses, ≥3 losses, consecutive vs. non-consecutive losses, and inclusion of biochemical pregnancies) was specifically recorded. Because vitamin D thresholds differed across studies, extracted data also included the definition of deficiency or insufficiency used by each article (e.g., <20 ng/mL, <30 ng/mL, or assay-specific reference ranges). Data extraction included study design, population characteristics, vitamin D assessment, genotyping methods, and clinical outcomes, while quality appraisal assessed study design suitability, sample size, methodological rigor, and statistical robustness. Due to notable heterogeneity in study designs, outcome definitions, and analytical approaches, a qualitative narrative synthesis was performed instead of a meta-analysis, ensuring a structured and transparent evaluation of the available evidence.

In this systematic narrative review, heterogeneous clinical, genetic, mechanistic, and translational studies were synthesized based on the quality appraisals of different domains rather than one tool of risk of bias. The clinical and genetic studies were evaluated based on design, sample size, case definition, measurement, adjustment for confounders, and reporting transparency, guided by principles derived from the Newcastle-Ottawa framework, whereas the mechanistic studies were appraised based on model relevance and biological plausibility. Risk of bias among the studies was categorically identified to guide interpretation. Considering the heterogeneity across the definition of RPL, populations, vitamin D cut-offs, genotyping techniques, endpoints, and study designs, meta-analysis and GRADE assessment were inappropriate; thus, the results were synthesized based on qualitative domains such as risk of bias, consistency, directness, precision, and biological plausibility.

## 3. Vitamin D Biology and the Vitamin D Receptor

**Figure 1:**
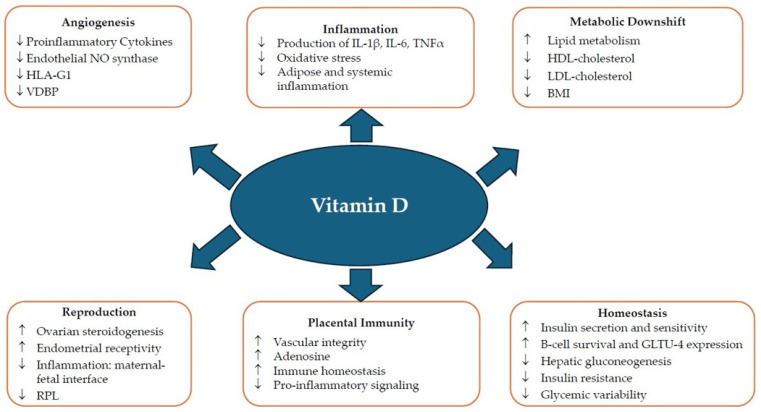
Pleiotropic actions of vitamin D relevant to RPL. Vitamin D modulates angiogenesis, lipid and glucose metabolism, and placental immunity by reducing inflammatory mediators, metabolic dysfunction, and natural killer (NK)-cell activity while enhancing ENTPD1–adenosine signaling. These coordinated effects support endometrial receptivity and the maintenance of early pregnancy.

Vitamin D is a secosteroid hormone produced in the skin or acquired through diet, then converted to its active form, 1,25-dihydroxyvitamin D. It regulates calcium homeostasis and has broad endocrine, immune, and metabolic effects [16,27]. These functions are mediated by VDR, a ligand-activated nuclear transcription factor similar to other steroid hormone receptors [28]. When a ligand binds, VDR heterodimerizes with the retinoid X receptor and attaches to vitamin D response elements to control the expression of genes involved in immune tolerance, decidualization, trophoblast invasion, cellular differentiation, and metabolic activity [29,30]. Human-tissue-expression studies have demonstrated VDR presence in the endometrium, placenta, immune cells, and metabolic tissues [31]. Its expression appears to be dynamic across the menstrual cycle and may increase during the progesterone-dominant secretory phase, consistent with a role in endometrial receptivity and decidualization. Based largely on mechanistic laboratory and ex vivo evidence, vitamin D signaling is hypothesized to integrate endocrine, immune, and metabolic pathways essential for implantation and early pregnancy homeostasis (Figure 1) [32].

The *VDR* gene, located on chromosome 12q13.11 and spanning 75 kb, contains over 4000 reported single-nucleotide polymorphisms (SNPs) [33], although only 200–300 have a minor allele frequency of ≥1%, and a smaller subset has been consistently examined clinically. Promoter variants such as Cdx2 and GATA influence transcription regulation [34], exonic variants like FokI (rs2228570) alter the start codon and modify receptor activity [35], and intronic polymorphisms, including BsmI (rs1544410), ApaI (rs7975232), and TaqI (rs731236), may impact mRNA stability or reflect LD with functional sites [34,35]. Genetic variation in *VDR* contributes to individual differences in vitamin D-dependent endocrine and immune regulation [36], with population-specific haplotypes associated with diverse immune, metabolic, and reproductive phenotypes, often through interactions with vitamin D status rather than isolated genetic effects [27,37]. Vitamin D deficiency is increasingly recognized as a systemic endocrine disorder linked to inflammation, immune dysregulation, and adverse reproductive outcomes [38], and ligand-activated VDR signaling is vital for immune tolerance at the maternal–fetal interface [16,21].

## 4. Vitamin D Deficiency and Immune-Metabolic Mechanisms in RPL

**Figure 2:**
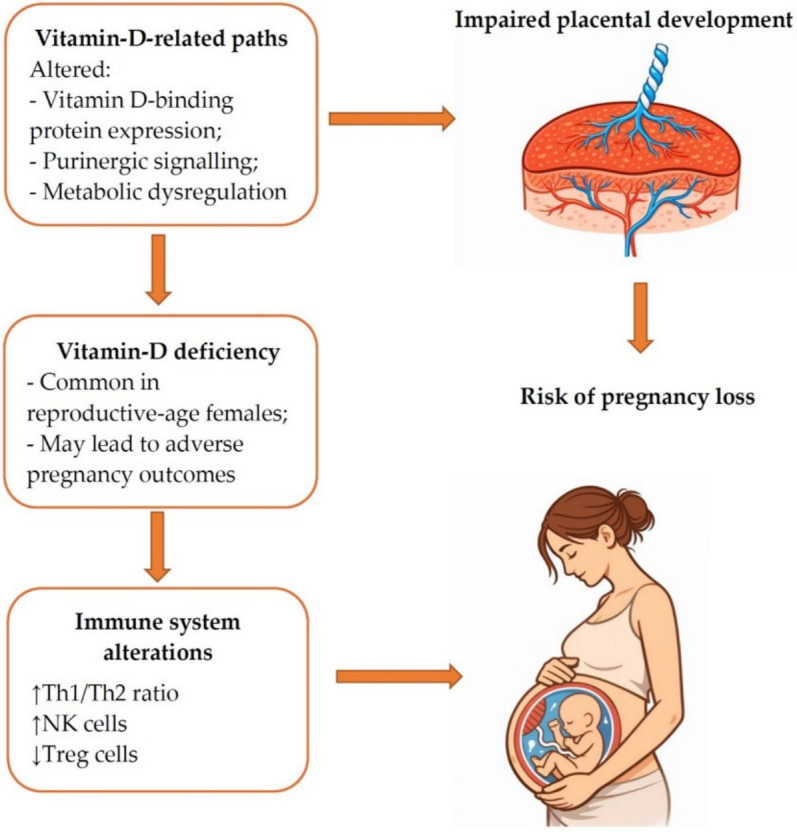
Overview of vitamin D deficiency and its role in RPL. Deficiency across all trimesters is linked to immune imbalance, marked by elevated NK cells, Th1/Th2 ratios, and reduced Treg function and impaired maternal–fetal tolerance. Genetic variation in *VDBP* affects bioavailability and placental development, while metabolic dysregulation and disrupted purinergic signaling further compromise implantation and trophoblast function. These converging pathways contribute to increased susceptibility to RPL.

Unless otherwise stated, vitamin D deficiency is defined in this review as serum 25-hydroxyvitamin D [25(OH)D] <20 ng/mL (<50 nmol/L), and is highly prevalent among reproductive-aged women, affects all trimesters, and is linked to adverse outcomes, including RPL (Figure 2) [39]. Some reproductive studies used higher thresholds (e.g., <30 ng/mL) to define insufficiency or suboptimal status. Beyond its classical role in calcium regulation, vitamin D exerts essential immunomodulatory effects required for implantation and placentation [40]. In human observational cohorts, women with RPL and low vitamin D frequently have been reported to exhibit immune disturbances, including elevated natural killer (NK)-cell activity, higher Th1/Th2 cell ratios, and reduced T-regulatory Treg) cell function [41]. These changes are complemented by laboratory studies showing the immunomodulatory effects of vitamin D on these pathways. Whereas experimental studies and cell-based models indicate that vitamin D also influences angiogenesis, lipid and glucose metabolism, placental immune balance, and inflammatory signaling, and upregulates ENTPD1-mediated adenosine pathways involved in immune regulation, human cohort data primarily support associations between deficiency states and adverse reproductive outcomes [40,41,42,43]. Because vitamin D signaling supports immune tolerance, metabolic stability, and vascular adaptation at the maternal–fetal interface, reduced ligand availability or impaired *VDR* function can promote inflammation, inadequate decidualization, and defective trophoblast invasion. Deficiency is further associated with autoimmune and inflammatory disorders in pregnancy [41,42], underscoring its role in maintaining immune–endocrine equilibrium [40,44,45,46,47,48]. Vitamin-binding protein (VDBP), highly expressed in placental tissue, also regulates vitamin D availability; genetic variants affecting VDBP may contribute to pregnancy complications, including RPL [23,45,46,49].

Graph evidence shows that vitamin D-related pathways intersect with metabolic and immune mechanisms involved in RPL [50,51]. Metabolic dysfunction can worsen the effects of low vitamin D or impaired VDBP activity [50,51]. Purinergic signaling via ENTPD1 (CD39) and NT5E (CD73) is essential to produce adenosine, a key mediator of immune suppression, vascular adaptation, and trophoblast function in early pregnancy [52,53]. Disruption of these pathways, especially when amplified by vitamin D deficiency or VDR dysfunction, can increase inflammation and hinder placental development. Mitochondrial energy shortages further impair implantation and embryonic growth [50,53]. Overall, vitamin D deficiency and VDBP genetic variation can cause immune and metabolic imbalance, negatively affect implantation and placentation, and raise the risk of RPL.

## 5. Current Evidence Linking VDR Polymorphisms to RPL

Current evidence suggests that VDR variants may modify RPL susceptibility but should be discussed in the context of vitamin D signaling linked to maternal immune system regulation, metabolic pathways, and endometrial receptivity [19,35]. A major concern is heterogeneity in RPL definitions between ASRM, ESHRE, and RCOG, causing inconsistency in epidemiological data [2,3,4]. The broader criteria of ASRM and ESHRE (≥2 losses) potentially capture a more heterogeneous phenotype than stricter definitions of RCOG requiring ≥3 consecutive clinically recognized losses. Current evidence suggests that the *VDR* gene variants may influence RPL susceptibility, but associations are inconsistent across studies (Table 1) [54]. In addition, most available studies enrolled relatively small cohorts (<200 participants), limiting the power to detect modest genetic effects and increasing susceptibility to false-positive or ancestry-specific findings.

It is believed that reduced signaling potential by *VDR* genes has a profound effect on the tolerance ability of the maternal/immune system, as well as decidual activity in vitamin D deficiency conditions [16,21]. This is important in order to comprehend the numerous links between the *VDR* gene polymorphism and certain genetic markers among different populations [32]. Of all the studied loci, FokI appears to have received the most attention, generating more significant results compared to BsmI, ApaI, or TaqI sites.

## 6. Functional vs. Regulatory VDR Variants

### 6.1. FokI (rs2228570) and Its Contribution to RPL Susceptibility

**Figure 3:**
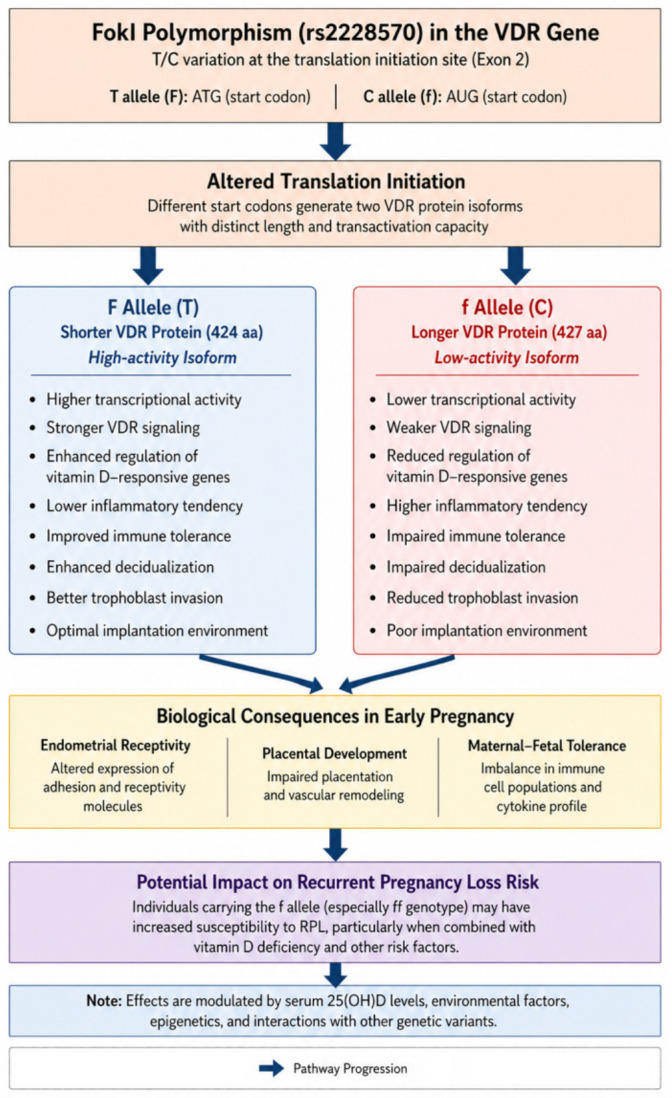
Functional implications of the FokI polymorphism (rs2228570) in RPL. The variant modifies the vitamin D receptor (VDR) start codon, resulting in two protein isoforms: a shorter F-allele-encoded receptor with higher transcriptional activity and a longer, less active isoform. These isoforms are shown alongside a schematic DNA element to illustrate their regulatory roles. The increased activity of the shorter isoform supports key processes in early pregnancy, including maternal–fetal immune tolerance and trophoblast function, invasion, and endometrial receptivity. Reduced VDR signaling, particularly in the context of vitamin D deficiency, may impair these pathways and contribute to heightened RPL susceptibility. Created using Adobe Illustrator (version 28.5, Adobe Inc., San Jose, CA, USA).

Vitamin D signaling is one of the key factors important for an appropriate endometrial receptivity and maternal–fetal immune balance and tolerance. Thus, a growing interest is observed in the field of investigations on the *VDR* gene variants in pregnancy loss. Among these, FokI (rs2228570) is the only polymorphism that alters *VDR* translation, generating protein isoforms with different activities (Figure 3) and making it a plausible susceptibility locus for idiopathic RPL, especially in populations with vitamin D deficiency [25,55]. However, evidence linking FokI to RPL varies across studies and ethnic groups. Slovenian and Croatian cohorts reported increased RPL risk with the maternal C allele and the C/C genotype [25], and similar associations were observed in an Iranian case–control study supported by in silico predictions of reduced VDR stability [56]. In contrast, data from Polish studies did not reveal associations with FokI but identified other VDR variants and protective haplotypes [26]. A Chinese cohort reported fewer F alleles and fewer FF/Ff genotypes among RPL cases, suggesting a reduced risk, especially in vitamin D-deficient women [57]. No link was found in the Indian Bengali population [58]. These differences probably stem from variations in ethnic allele frequencies, differing RPL definitions, and methodological factors such as sample size, genetic models, and covariate adjustments [2,7].

Overall, most RPL case–control studies assessing FokI reported at least one significant association at the allele, genotype, or haplotype level, while others found no effect, suggesting a potential signal without consistent replication across populations. Confidence in the clinical evidence remains low to moderate, as many studies were small, single-center, and methodologically heterogeneous. Positive findings were weighed according to study quality, with greater caution applied to underpowered or unadjusted analyses. Given frequent sample sizes below 100–200 cases, both positive and null results should be interpreted conservatively.

Functionally, FokI introduces a T > C substitution in exon 2, generating two VDR isoforms that differ by 3 amino acids but exhibit distinct transcriptional activity [65]. The shorter F-allele isoform has higher transactivation capacity than the less active f-allele isoform (Figure 3). Because VDR signaling regulates immune polarization, Treg-cell induction, decidualization, trophoblast invasion, progesterone-responsive endometrial receptivity, and placental angiogenesis [38,40,66], these functional differences are relevant to early pregnancy. Reduced VDR expression in decidual and chorionic tissues has been reported in some women with RPL. These *ex vivo* findings are consistent with impaired vitamin D signaling, although causality has not been established [42,67]. Notably, the impact of FokI appears amplified under vitamin D deficiency, consistent with gene–environment interactions and the mechanisms summarized in Figure 3.

Overall, FokI is the most frequently studied *VDR* variant in RPL with a strong biological rationale as it alters the translation initiation site of the receptor (Figure 3), however, associations vary by population and study design. Discrepancy in findings may also be explained by different case definition standards because some of the studies employed a wider range of RPL standards, involving patients with diverse etiologies, whereas some were limited to recurrent phenotype conditions only. Present findings indicate contextual influences mediated through ethnicity, genetic structure, and vitamin D status [25]. Due to small sample sizes and differences in methodology, further research with well-defined RPL standards, multi-ethnic study groups, haplotyping, and vitamin D levels would be necessary.

### 6.2. Association of the Non-Coding BsmI, ApaI, TaqI VDR Variants with RPL

In parallel with the functional FokI variant, the commonly studied BsmI (rs1544410), ApaI (rs7975232), and TaqI (rs731236) polymorphisms map to the 3′ region of the *VDR* gene. Support for such non-coding mutations is limited by small sample sizes in several studies using a single-center sample size with low statistical power to detect association. While they do not affect the amino acid sequence of the gene, these polymorphisms have very strong LD with each other as well as other 3′-UTR/polyA region mutations, creating regulatory haplotypes affecting stability, splicing, or mRNA expression [26]. As with FokI, this means that the biologically relevant unit of association is often the haplotype, or an unmeasured causal variant it tags, helping explain inconsistent findings across populations [68,69].

In contrast to the functionally characterized FokI SNP, relationships with BsmI, ApaI, and TaqI show considerable heterogeneity, indicating that they are likely to represent regulatory effects acting through specific patterns of LD in distinct populations [70,71]. Regulation of VDR expression is capable of affecting vitamin D-regulated transcriptional events critical to decidualization, cytokine regulation, and trophoblastic invasion, all of which are responsive to hormone ligand availability and immune–metabolic stress [21,31]. The reported relationships between these SNPs and RPL may arise from situations in which vitamin D deficiency, inflammation, or metabolic stress play a role, and may be due to factors related to LD structure and population ancestry rather than any functional effects of these SNPs themselves [15]. These discrepancies may reflect differences in LD architecture, ancestry, study design, or environmental context, although definitive explanations remain uncertain [68].

Consistent with the reports on FokI, multi-marker haplotype analyses of TaqI-ApaI-BsmI, often studying FokI, may better explain the effects and role in RPL risks compared to single-marker tests [59]. In the Polish cohort, several haplotypes were more common in controls and interpreted as protective, including a four-marker haplotype that remained significant after permutation testing [26]. This supports a model in which combinatorial 3′ variation more accurately reflects functional differences in VDR transcript regulation than any individual SNP, complementing the mechanistic framework established for FokI.

#### 6.2.1. BsmI (rs1544410)

The BsmI polymorphism is the most extensively studied non-coding VDR variant in RPL, yet reported associations vary widely across populations. The BB genotype was linked to increased RPL risk in Egyptian women [60], and the CC genotype and C allele were more frequent among Slovenian and Croatian women with RPL [25,72]. In Serbian women, BsmI was associated with infertility, with specific BsmI-linked haplotypes conferring either increased susceptibility or protection depending on infertility subtype [59]. A broader population-level signal has also been noted, with BsmI showing associations with infertility primarily in Asian cohorts [62]. Vitamin D deficiency may further modify these relationships, suggesting a gene–environment interaction [73].

Amongst all studies, the most extensive study regarding the 3′ end of the locus is one on the Polish population, in which the distribution of BsmI genotypes and alleles showed differences between the cases and controls, although individual associations became insignificant when adjusted for multiple comparisons [26]. Other small studies, such as one conducted on an Iraqi population, found associations, but their small sample sizes and possible confounders limit their conclusions [61]. In general, BsmI shows some consistent associations, but not enough to be called reliable in different populations, and is possibly a marker of an entire regulatory haplotype rather than a causal mutation [26]. Consistent with this opinion are the findings that haplotypes carrying BsmI were more frequent in controls in a Polish study [26].

#### 6.2.2. ApaI (rs7975232)

Evidence for a link between the ApaI (rs7975232) polymorphism and RPL remains inconsistent. A small case–control study reported significant genotype differences between the RPL cases and controls [56], while an even smaller Iraqi study found only marginal associations [61]. Maternal ApaI-containing *VDR* haplotypes seem to be protective, as they are more common in healthy multiparous women [26]. A similar negative association with pregnancy loss was seen in Iranian women when ApaI was evaluated alongside other *VDR* markers, though with typical candidate-gene limitations [56]. A recent meta-analysis also indicated a protective effect, but only under specific genetic models, showing that ApaI associations depend on both the model and the population [62].

These observations are in line with the role of ApaI within the larger context of the 3′ regulatory haplotype, where phenotypic expression is dependent on vitamin D levels, immune environment, and specific ancestry-based linkage disequilibrium [74]. Thus, it would be best to regard ApaI polymorphism as a context-dependent biomarker, which usually comes up negative when considered in isolation.

#### 6.2.3. TaqI (rs731236)

TaqI is a synonymous exon-9 VDR variant defined by a T→C substitution at codon 352, which may influence transcriptional or translational efficiency. Maternal TaqI variation has been linked to altered risks of pregnancy complications [75], including preterm birth, low birth weight [76], and RPL [25,26]. Only a limited number of studies have examined TaqI specifically in RPL, and findings remain mixed. Among Sudanese women, the TaqI variant was observed more frequently in cases (97.7%) than in controls (14%), suggesting a strong association, though the small sample size and lack of an odds ratio limit interpretation [63]. A recent meta-analysis of female infertility, which included four miscarriage studies across diverse populations, reported increased susceptibility associated with the TaqI variant under multiple genetic models, but did not provide RPL-specific pooled estimates [62]. A literature review similarly identified TaqI (rs731236) as one of the most frequently studied VDR variants in idiopathic RPL, yet lacked sample sizes, ethnic details, or genotype-specific effects, restricting conclusions [55].

In contrast, no significant association between TaqI and RPL was observed in Croatian [26] or Slovenian-Croatian cohorts [25], despite clear FokI-related susceptibility signals [25]. Because TaqI is a synonymous SNP, any observed effect is more plausibly attributable to linkage disequilibrium with functional 3′ regulatory elements rather than a direct coding impact. Clarifying its contribution to RPL will require well-powered case–control studies with standardized genotyping and complete reporting of allele/genotype frequencies and effect estimates.

### 6.3. Population-Specific Patterns

Evidence across studies indicates that unlike the functional FokI variant [25], associations involving the BsmI, ApaI, and TaqI polymorphisms are inconsistent, population-specific, and strongly shaped by contextual factors [62,77]. Because these non-coding variants are located primarily in regulatory regions of the *VDR* gene and do not directly alter protein structure, their observed effects likely reflect regulatory mechanisms, such as LD with functional elements, ancestry-related genomic differences [61], and gene-environment interactions involving vitamin D status [55], that contribute to the heterogeneity reported across populations [78]. From a clinical practice perspective, these polymorphisms should not be interpreted as separate genetic markers for risk of pregnancy loss. However, these polymorphisms are important to consider when analyzing *VDR* gene haplotypes [61,62] and are evaluated alongside serum vitamin D levels [55]. This is highly valuable in a precision-medicine approach, as regulatory *VDR* gene variations, when analyzed with vitamin D status, may help identify women who are particularly vulnerable to the adverse reproductive consequences [55,62].

### 6.4. Notes on the Associations of the Non-Coding BsmI, ApaI, TaqI VDR Variants, and RPL

Evidence supports FokI (rs2228570) as a functional *VDR* variant influencing reproductive outcomes. However, the assumption that BsmI, ApaI, and TaqI are strictly noncoding and nonfunctional is not well-substantiated, as sequencing data [63] and in silico analyses [56] indicate that several 3′ variants, particularly TaqI, may affect splicing or protein stability. These possibilities are rarely considered, and most studies treat BsmI/ApaI/TaqI as nonfunctional markers simply because their associations vary across ancestry groups and reflect clear LD patterns. Such interpretations often rely on oversimplified single-SNP models, especially when environmental factors like vitamin D status are not accounted for.

Because these loci are in LD [79], the allele of “risk” for one population may tag a different regulatory element in another [62], meaning that discordant single-marker findings often reflect study-design limitations rather than true null effects. Haplotype-based analyses better capture underlying biology, as demonstrated previously [26], which identified the protective TTGT haplotype (0.09 vs. 0.017, *p* = 0.0024), and by multi-SNP reproductive studies [76,77]. Collectively, these data suggest that treating BsmI/ApaI/TaqI as isolated markers obscures meaningful regulatory structure within the 3′ *VDR* region.

### 6.5. Associations Beyond FokI, BsmI, ApaI, and TaqI in RPL

Evidence for *VDR* variants beyond the four canonical SNPs in RPL is extremely limited. Although several “non-core” variants could plausibly affect *VDR* regulation at the maternal–fetal interface, they remain largely untested in robust RPL cohorts. The only moderately documented noncanonical SNP (rs10735810) shows no clear association, underscoring that current data are sparse, inconsistent, and insufficient to support any conclusions.

#### 6.5.1. The Cdx2 Polymorphism (rs11568820, A/G)

Cdx2 is located within a CDX2 transcription-factor binding site in the upstream *VDR* regulatory region, and its variants are considered functional because allele-specific TF binding may influence tissue-specific *VDR* expression. Mechanistic studies in non-placental systems support the idea of differential CDX2 affinity and changes in promoter activity [64]. Since vitamin D plays roles in decidualization, immune tolerance, and placental development, these regulatory effects are biologically plausible. However, direct evidence linking the Cdx2 rs11568820 variant to altered RPL risk remains limited. The only well-characterized case–control study, conducted in Slovenian/Croatian cohorts, found no association between maternal Cdx2 genotypes and RPL, despite a clear FokI-related susceptibility signal in the same population [26].

Cdx2 remains a functionally plausible but clinically unconfirmed RPL locus. The negative European findings do not exclude population-specific effects, small effect sizes, vitamin-D-dependent gene–environment interactions, or context-specific regulation across decidual, trophoblast, and immune tissues. Further studies are needed to clarify whether Cdx2 contributes to RPL risk in specific genetic or environmental contexts.

#### 6.5.2. Poly(A) Length Polymorphism (rs17878969; 3′ UTR)

Since it is in strong LD with the BsmI/ApaI/TaqI block, which may influence post-transcriptional regulation, the polyadenylation-related 3′-UTR polymorphism (rs17878969) was recently proposed to be closely linked to BsmI, ApaI, and TaqI [26]. Although the rs17878969 variant was suggested to impact mRNA stability, functional assays showed no differences in stability between common *VDR* 3′ UTR haplotypes [80]. No further studies have directly examined the association of rs17878969 with RPL, and any observed association is probably due to LD with nearby variants rather than an independent effect [26,81]. Overall, the current evidence indicates that rs17878969 is more a marker of the wider 3′ haplotype structure than an independent RPL risk factor, underlining the importance of future research involving direct genotyping and expression-based analyses in decidua or placenta.

#### 6.5.3. Tru9I (rs757343; intron 8)

The Tru9I polymorphism (rs757343), an intronic A/G variant in *VDR* intron 8, remains unstudied in RPL and likely acts as a passive marker of the BsmI-ApaI-TaqI 3′ haplotype block, rather than as an independent functional locus [82]. Although it has been evaluated in limited non-RPL reproductive contexts, such as maternal–neonatal anthropometry [82] and is occasionally discussed in relation to vitamin D-related or immune-mediated phenotypes, it is consistently absent from major RPL studies and recent reproductive–endocrine reviews that focus on the canonical FokI, BsmI, ApaI, and TaqI variants [83]. While intron 8 offers theoretical regulatory potential, there is no functional or association data supporting a role for Tru9I in RPL. Its relevance remains speculative and warrants targeted investigation in multi-ethnic RPL cohorts, including analyses stratified by vitamin D status and models incorporating immune signatures relevant to implantation.

## 7. Gene–Environment Interactions

Although several studies suggest effect modification by vitamin D deficiency, most relied on stratified analyses rather than formal genotype × environment interaction terms, limiting causal inference [84]. Only a few investigations examined specific *VDR* variants, circulating 25(OH)D levels, and overall RPL risk [85,86], restricting the ability to evaluate gene–environment interactions. We support the hypothesis that *VDR* polymorphisms are not direct causal factors but contribute to RPL risk within the broader context of vitamin D status and the specific variants examined [26,55]. This highlights the importance of population differences in genetic studies and suggests that VDR-related susceptibility becomes clinically meaningful primarily when vitamin D levels are inadequate.

Experimental molecular studies indicate that vitamin D functions through ligand-activated VDR, and genotype-related differences in *VDR* expression or function may be lessened when circulating 25(OH)D levels are adequate [36]. Conversely, deficiency can reveal the effects of inadequate VDR signaling in pathways vital for early pregnancy, including decidualization, trophoblast invasion, and maternal immune tolerance [31,87]. Ethnicity-specific LD and haplotypes further influence the effect of *VDR* genotypes [25,63,88]. Since VDR signaling affects decidual NK cell activity, Treg-cell differentiation, and cytokine balance, genetic variation likely impacts these processes [23], although direct functional evidence in RPL is limited and should be considered hypothesis-generating.

Available clinical and epidemiological data support this interactive model, in which genotype effects may become more evident in vitamin D-deficient women, most commonly using serum 25(OH)D thresholds <20 ng/mL (<50 nmol/L), although some studies applied higher cutoffs; however, formal interaction analyses remain limited [14,15,57]. Additional findings indicate that VDR variant effects may depend on environmental factors such as sunlight exposure and dietary vitamin D intake [89] and may be shaped by combinations of *VDR* polymorphisms rather than single loci [26,90]. Overall, *VDR* genotyping alone has limited clinical value for RPL risk assessment. Meaningful interpretation requires integrating *VDR* variation with vitamin D status, environmental exposures, and ethnicity-specific factors [91]. Because most studies did not include genotype × vitamin D interaction terms, existing stratified associations should be considered exploratory rather than confirmatory.

## 8. Clinical Implications and Translational Relevance

**Figure 4:**
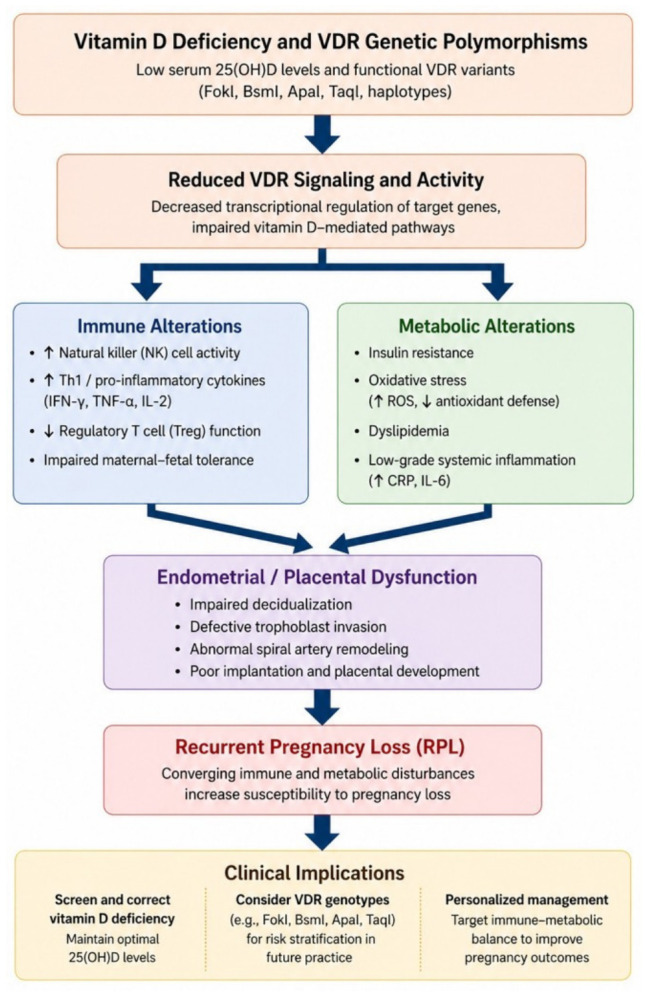
Proposed pathway linking vitamin D deficiency and vitamin D receptor (VDR) polymorphisms to recurrent pregnancy loss (RPL). Figure legend: Low circulating vitamin D and reduced-function VDR variants may attenuate receptor signaling, leading to immune imbalance, metabolic disruption, impaired decidualization, abnormal trophoblast invasion, and defective placentation, thereby increasing RPL risk. Clinical implications include vitamin D screening and repletion, with future potential for genotype-based risk assessment. Created using Adobe Illustrator (Adobe Inc., San Jose, CA, USA).

Vitamin D status is increasingly seen as a potentially modifiable factor in reproductive medicine. Although international RPL guidelines do not generally recommend universal routine serum vitamin D testing as part of the standard basic infertility evaluation, and thus routine VDR genotyping [2,3,4], the current evidence suggests that assessment and correction of vitamin D deficiency may be reasonable in women with RPL or repeated implantation failure [14,57]. This is especially relevant in populations where vulnerable *VDR* genotypes such as FokI are common [63,92], and areas with widespread vitamin D deficiency caused by limited sun exposure or diet, but their current clinical utility has not been established [26,57,63]. These interactions may be particularly relevant during the luteal phase, when progesterone-dependent endometrial receptivity and vitamin D responsiveness are both required for successful implantation. Taken together, mechanistic laboratory findings and human observational data suggest the relevance of gene–environment interactions when assessing vitamin D status in RPL and recommend supplementation if deficiency coexists with at-risk *VDR* genotypes [20,57]; however, direct clinical trial evidence in women with RPL remains limited. To bring such strategies into clinical practice, stronger evidence from larger studies that include both *VDR* genotyping and serum vitamin D testing is needed to determine whether genotype-based optimization improves reproductive outcomes [26,86,93]. The proposed pathways through which vitamin D deficiency and *VDR* genetic variation may contribute to RPL, together with their potential clinical applications, are summarized in Figure 4.

This distinction is important: vitamin D testing should not be presented as a routine first-line infertility investigation, but rather as an adjunctive assessment in selected patients or reproductive-failure phenotypes. Beyond traditional candidate-gene variants, genome-wide approaches may discover additional RPL-related loci [93], which will require functional validation within pathways connecting *VDR* variation to pregnancy loss [26,56]. Clinically, interpretation of vitamin D status should consider local guideline thresholds, assay methodology, seasonal variation, and whether deficiency (<20 ng/mL) or insufficiency (<30 ng/mL) definitions are being applied. Well-controlled prospective studies examining *VDR* genotypes alongside circulating vitamin D levels and RPL outcomes are crucial to confirm or disprove causality. Genotype-stratified randomized trials of vitamin D supplementation in women with RPL could determine whether targeted intervention enhances reproductive success [86,94] while also identifying optimal dosing, timing, duration, and the roles of nutrients such as calcium and magnesium that influence vitamin D metabolism.

## 9. Future Research Directions

The main message of this review is that meaningful advances necessitate moving beyond isolated candidate-gene studies toward integrated models that jointly consider genetic background, vitamin D status, immune function, and metabolic profiles. Reported metabolic comorbidity rates, including insulin resistance, are difficult to compare across studies because of non-standardized diagnostic thresholds and variable adjustment for obesity and PCOS. Among the studied variants, FokI shows the most consistent signal, although findings remain population-dependent and limited by small sample sizes, heterogeneous RPL definitions, and variable methodological quality across studies.

Further research should take advantage of larger homogeneous patient groups for supporting formal criteria of evidence, such as GRADE and quantitative heterogeneity analysis, along with moving from SNPs alone toward more complex interaction-related modeling, capturing the endocrine, metabolic, and immunologic complexities of recurrent pregnancy loss. Adequately powered multicenter case–control cohorts and meta-analytic collaborations should aim to address issues associated with small single-center case series. Further progress requires modern standardized definitions of RPL with the proper reporting of biochemical pregnancy and assisted reproductive technique conception status, along with distinguishing primary and secondary cases, standardization of vitamin D measurement, and adequately powered multicenter, multiethnic cohorts. Genetic and sequencing techniques will be necessary for capturing common and rare genetic variants within the VDR signaling pathway [95].

Several other limitations need to be mentioned. Publication bias is possible due to smaller candidate genes that find positive associations being more likely to be published than negative findings. Another limitation is the restriction of the search strategy to indexed literature written in English, which could have resulted in overlooking regional literature. Furthermore, the lack of complete genotype data and effect estimates, as well as standardization of confounding variables, also posed problems in cross-comparison between studies. A key priority is formally modeling genotype and vitamin D interactions using baseline 25(OH)D levels, longitudinal designs, and genotype-stratified trials to test whether supplementation efficacy differs by *VDR* genotype and vitamin D status [57,91]. Mechanistic advances will require single-cell transcriptomics, multi-omics integration, and functional assays in the decidual, trophoblast, and immune systems to define how regulatory and functional variants, such as FokI, reshape implantation pathways [26,96]. Incorporating epigenetics, microbiome profiling [97], and polygenic models within international biobanking consortia [98] will accelerate precision-medicine strategies for RPL prevention.

### Strengths and Limitations

The manuscript contains several strengths, including a thorough and profound analysis of the available literature on the subject, as well as the attempt to combine molecular, clinical, and epidemiological approaches to exploring VDR and the *VDR* gene polymorphisms and their involvement in RPL pathogenesis. The literature for a wide period covered in the review provides an opportunity to include both pioneering works and those published relatively recently. At the same time, some limitations of this review should also be taken into account. In particular, as the manuscript is written in a narrative format and lacks a systematic approach to evaluating the quality of studies used, there is a possibility of selection bias. Furthermore, due to the heterogeneity of the included research, generalizations cannot be made.

## 10. Conclusions

Overall, current evidence from experimental models suggests that vitamin D deficiency and *VDR* gene variation may contribute to RPL risk through immune and hormonal pathways essential for implantation and early placentation, while human observational studies suggest possible clinical associations between vitamin D/VDR pathways and RPL. FokI has the most evidence and the clearest functional rationale, primarily in vitamin D deficiency or related adverse biological contexts, although associations with RPL remain heterogeneous and not definitively established, being constrained by the predominance of small studies and inconsistent replication across populations. Clinically, correcting vitamin D deficiency appears reasonable and potentially beneficial, particularly in women with RPL or other reproductive-failure phenotypes, whereas *VDR* genotyping offers no meaningful value for RPL risk assessment. The newly added mechanistic framework (Figure 4) also highlights how integrated endocrine, immune, and genetic profiling may support future personalized prevention strategies. The cycle-dependent and progesterone-regulated nature of endometrial VDR expression further supports a temporally specific role for vitamin D signaling during implantation. At present, routine *VDR* genotyping is not supported for clinical care, and further well-designed prospective studies are needed to clarify causality and therapeutic relevance. These conclusions should be interpreted in the context of narrative review methodology, potential publication bias, and variability in reporting quality across the underlying literature. A more integrated endocrine–immune–metabolic framework may ultimately support individualized strategies for RPL prevention.

## Figures and Tables

**Table 1:** Summary of reported associations between the *VDR* gene polymorphisms and RPL, with population-wise background, prevalence estimates, and supporting references.

*VDR* Polymorphism	Mode of Association with RPL	Populations/Settings	RPL Incidence/Prevalence in Studied Population *	Adjustment for Confounders	Vitamin D Interaction Tested	References
FokI (rs2228570)	Mostly positive (risk-increasing), allele- and genotype-dependent	Predominantly Asian and Middle Eastern cohorts; inconsistent in European populations	1–5%; clinic cohorts enriched	Partial; age and BMI most common; limited metabolic/endocrine adjustment	Rare; mostly stratified analyses rather than formal interaction terms	[25,55,56,57,58]
BsmI (rs1544410)	Inconsistent (risk-increasing, protective, or null)	Sporadic population-specific associations; largely null in European cohorts	1–3% in registry-based European estimates	Limited; many analyses unadjusted or minimally adjusted	No	[26,59,60,61]
ApaI (rs7975232)	Inconsistent or null	Occasional ethnicity-specific associations	1–5%; similar to regional RPL baseline estimates	Limited; adjustment uncommon	No	[26,56,61,62]
TaqI (rs731236)	Mostly null; occasional weak associations	Isolated findings in small cohorts; no consistent population pattern	Insufficient study-specific estimates but similar to regional baseline	Rare	No	[25,26,62,63]
Cdx2 (rs11568820)	Suggestive but insufficient evidence	Few exploratory studies, mainly Asian populations	1–5%; no robust population-specific estimates available	Variable; generally small sample sizes	No	[26,64]

* Intended to provide contextual background rather than direct cross-study comparisons, given that RPL prevalence varies substantially according to diagnostic criteria and study design.

## Data Availability

Data sharing is not applicable. No new data were created or analyzed in this study.

## Abbreviations

The following abbreviations are used in this manuscript:

25(OH)D	25-hydroxyvitamin D
1,25(OH)_2_D	1,25-dihydroxyvitamin D
ASRM	American Society for Reproductive Medicine
BMI	Body mass index
CI	Confidence interval
DBP	Vitamin D-binding protein
ESHRE	European Society of Human Reproduction and Embryology
GWAS	Genome-wide association study
IR	Insulin resistance
LD	Linkage disequilibrium
MAF	Minor allele frequency
NK cells	Natural killer cells
OR	Odds ratio
RCOG	Royal College of Obstetricians and Gynecologists
RPL	Recurrent pregnancy loss
RXR	Retinoid X receptor
SNP	Single-nucleotide polymorphism
Th1/Th2	T-helper 1/T-helper 2 immune pathways
UTR	Untranslated region
VDR	Vitamin D receptor
